# Mild Choline Deficiency and MTHFD1 Synthetase Deficiency Interact to Increase Incidence of Developmental Delays and Defects in Mice

**DOI:** 10.3390/nu14010127

**Published:** 2021-12-28

**Authors:** Karen E. Christensen, Olga V. Malysheva, Stephanie Carlin, Fernando Matias, Amanda J. MacFarlane, René L. Jacobs, Marie A. Caudill, Rima Rozen

**Affiliations:** 1 Departments of Human Genetics and Pediatrics, McGill University, Montreal, QC H3A 0C7, Canada; karen.christensen@mail.mcgill.ca; 2 The Research Institute of the McGill University Health Centre, Montreal, QC H4A 3J1, Canada; 3 Division of Nutritional Sciences, Cornell University, Ithaca, NY 14853, USA; ovm4@cornell.edu (O.V.M.); mac379@cornell.edu (M.A.C.); 4 Division of Human Nutrition, Department of Agricultural, Food and Nutritional Science, University of Alberta, Edmonton, AB T6G 2E1, Canada; carlin@ualberta.ca (S.C.); rjacobs@ualberta.ca (R.L.J.); 5 Nutrition Research Division, Health Canada, Ottawa, ON K1A 0K9, Canada; fernando.matias@hc-sc.gc.ca (F.M.); amanda.macfarlane@hc-sc.gc.ca (A.J.M.); 6 Department of Biology, Carleton University, Ottawa, ON K1S 5B6, Canada

**Keywords:** MTHFD1, choline, folate, embryonic development, birth defects, developmental defects, developmental delay

## Abstract

Folate and choline are interconnected metabolically. The MTHFD1 R653Q SNP is a risk factor for birth defects and there are concerns that choline deficiency may interact with this SNP and exacerbate health risks. 80–90% of women do not meet the Adequate Intake (AI) for choline. The objective of this study was to assess the effects of choline deficiency on maternal one-carbon metabolism and reproductive outcomes in the MTHFD1-synthetase deficient mouse (*Mthfd1S*), a model for MTHFD1 R653Q. *Mthfd1S*^+/+^ and *Mthfd1S*^+/−^ females were fed control (CD) or choline-deficient diets (ChDD; 1/3 the amount of choline) before mating and during pregnancy. Embryos were evaluated for delays and defects at 10.5 days gestation. Choline metabolites were measured in the maternal liver, and total folate measured in maternal plasma and liver. ChDD significantly decreased choline, betaine, phosphocholine, and dimethylglycine in maternal liver (*p* < 0.05, ANOVA), and altered phosphatidylcholine metabolism. Maternal and embryonic genotype, and diet-genotype interactions had significant effects on defect incidence. Mild choline deficiency and *Mthfd1S*^+/−^ genotype alter maternal one-carbon metabolism and increase incidence of developmental defects. Further study is required to determine if low choline intakes contribute to developmental defects in humans, particularly in 653QQ women.

## 1. Introduction

Folate and choline are important nutrients in pregnancy because they are required for processes that are essential for embryonic development, including nucleotide and phospholipid synthesis, and methylation reactions [1,2,3]. 5,10-Methylenetetrahydrofolate (methyleneTHF) dehydrogenase–methenylTHF cyclohydrolase–formylTHF synthetase (MTHFD1) is an important enzyme that generates and interconverts one-carbon folates (Figure S1). The common R653Q variant (*MTHFD1* c.1958 G > A; rs2236225, ~20% QQ in European populations) compromises the synthetase activity of MTHFD1 [4] and has been identified as a risk factor for neural tube defects (NTD), heart defects, intrauterine growth restriction, and pregnancy loss [4,5,6,7]. In addition to its effects on folate metabolism, MTHFD1 R653Q has been found to alter choline metabolism in human studies [8,9,10,11,12]. Folate and choline metabolism intersect in the liver, the major site of both folate and choline metabolism. In the liver, both methylTHF and the choline derivative betaine are used to remethylate the toxic amino acid homocysteine to make methionine (Figure S1). The significance of this interrelationship is demonstrated by observations that inadequate folate or choline intakes can result in deficiency of the other nutrient in an attempt to maintain methyl metabolism [13,14,15,16].

The importance of adequate choline intake during pregnancy has become more recognized in recent years. Choline metabolism in the maternal liver supplies metabolites to the foetus that are essential for the support of brain development [3,17]. Low choline intakes have also been linked to NTD risk, even in folic acid fortified populations [18,19]. Choline deficiency in mice has been shown to cause NTDs in embryos in vitro [20] and heart defects in vivo [21]. The actual prevalence of choline deficiency in human populations is unknown, as an Estimated Average Requirement (EAR)/Recommended Dietary Allowance (RDA) for choline has yet to be established. However, it has been reported that 80–90% of women do not consume the recommended Adequate Intake (AI) for choline of 425 mg/d (450 mg/d during pregnancy) [22,23,24,25]. Choline intake may be of particular concern in women with the MTHFD1 R653Q variant, as QQ women have been hypothesized to have a higher dietary requirement for choline to sustain healthy pregnancies [26].

In earlier work, we generated the MTHFD1-synthetase deficient mouse (*Mthfd1S)* as a model for the 653QQ genotype. This mouse mimics the metabolic effects of the 653QQ genotype and is sensitive to both low and high folate diets with respect to negative reproductive outcomes at mid-gestation [4,27,28,29]. In this study, our objective was to assess the effects of choline deficiency on maternal choline metabolism and the incidence of embryonic delays and defects in the MTHFD1-synthetase deficient mouse. Using a choline-deficient diet containing one-third of the recommended amount for mice, which is proportional to the lower reported intakes of choline in women [24], we found interactions between low choline and MTHFD1-synthetase deficiency that affected the incidence of these reproductive outcomes.

## 2. Materials and Methods

### 2.1. Mice and Diets

Experiments were performed according to the Canadian Council on Animal Care guidelines and approved by the Animal Care Committee of the Research Institute of the McGill University Health Centre (protocol number 5585). *Mthfd1S* mice [27], 15 generations backcrossed onto BALB/cAnN (Envigo), were bred in-house and housed in specific pathogen-free facilities in a controlled environment (18–24 °C, 12-h light-dark cycle) with food and water ad libitum, in randomly-distributed cages in the same room. Unless noted, mice were fed standard chow (Teklad 2918, Envigo). Mice were genotyped as described [27].

Nulliparous female *Mthfd1S*^+/+^ (wild type (WT)) and *Mthfd1S*^+/−^ (heterozygous (HET)) mice were randomly assigned to control (CD) or choline-deficient diets (ChDD) (Envigo, Table S1) at four weeks of age. CD is a modification of standard amino acid–defined diets, as described [28,30]. The diets contain 1% succinylsulfathiazole to inhibit folate synthesis by intestinal flora. ChDD and CD differ only in choline bitartrate content: CD contains 2.5 g choline bitartrate/kg diet, whereas ChDD contains 0.8 g/kg. Mice were weighed when placed on diets, after four weeks on diets, at mating, and at sacrifice. Diet consumption was monitored by weighing the food weekly.

### 2.2. Timed Matings, Tissue and Embryo Collection

Nulliparous female WT and HET mice were fed CD or ChDD for 4–8 weeks before mating 1:1 with HET males (*n* = 16–18 per group; mean time on diet: 6.1 ± 0.1 weeks). Diet intervention duration did not vary significantly between diet/genotype groups, had no significant effects in the analysis of outcomes, and therefore was not included in the models for statistical analyses. The mice were maintained on the same diet throughout mating and pregnancy. This sample size was selected to be able to observe significant effects of diet and genotype on embryonic development, based on previous experiments with this strain [27,28,29]. The morning a vaginal plug was discovered was designated E0.5. At E10.5 the females were killed by CO_2_ asphyxiation under isofluorane anaesthesia. Blood was collected by cardiac puncture in EDTA-treated collection tubes. Plasma was separated by centrifugation at 3000 RCF for 7 min at 4 °C and snap frozen in dry ice. The liver and spleen were dissected, rinsed in cold phosphate buffered saline, weighed, and snap-frozen on dry ice. Frozen tissues were stored at −80 °C.

The uterine horns were removed, and implantation and resorption sites were counted. The number of eggs released was determined by counting corpora lutea on the ovaries. Embryo loss per litter was calculated as the percentage of resorption sites per total implantation and resorption sites. Embryos were dissected and evaluated for developmental stage as described [27,28,29] using established morphological markers. Typical E10.5 embryos had complete or near-complete closure of the posterior neuropore (tail tip), distinct forelimb buds, visible hindlimb and tail buds, a lens plate, a closed otic vesicle with a round or pointed shape, and 25–33 somites. Embryos ≥ 1 day behind their most developed littermate were considered to be developmentally delayed. Typical delayed embryos had an open posterior neuropore, no hindlimb or tail buds, no invagination of the optic vesicle, an open otic pit, and <24 somites (Figure S2). Embryonic crown-rump length (CR, Figure S3A) and somite number (Figure S4A) were determined when possible (extremely delayed, necrotic, or damaged embryos could not be assessed). To determine embryonic genotype and sex, DNA was extracted from the yolk sacs or if necessary, the entire embryo. Genotype was determined as in [27], while sex was determined as in [31]. Embryos were scored for the presence of developmental delays and defects by an individual blinded to diet/genotype group.

### 2.3. Measurement of Plasma and Liver Folates

Total folate was determined in the plasma and frozen liver using the *Lactobacillus casei* microbiological assay as in [32]. Liver folate concentrations were normalized to total protein content as determined using the Lowry assay [33].

### 2.4. Choline and Methylation Metabolite Measurement in Maternal Liver

Methionine, betaine, choline, glycerophosphocholine (GPC), phosphocholine, phosphatidylcholine (PtdCho), sphingomyelin, lysophosphatidylcholine (lyso-PtdCho), SAM, and S-adenosylhomocysteine (SAH) in frozen liver were measured using LC–MS [34,35,36,37].

### 2.5. Measurement of PtdCho:PtdE Ratio in Liver

A liver homogenate equivalent to 1 mg protein was extracted in the presence of phosphatidyldimethylethanolamine and batyl alcohol internal standards using a modified Folch method [38]. PtdCho, phosphatidylethanolamine (PtdE), triacylglycerol (TAG), and free cholesterol were measured by high performance liquid chromatography (HPLC) using a modification of the method of Abreu et al. as previously described [39,40].

### 2.6. Immunoblotting

Protein extraction from maternal liver and immunoblotting were performed as in [28] using primary antibodies specific for MTHFD1 [41], methyleneTHF reductase (MTHFR) [42], methionine synthase (MTR) (Proteintech 25896-1-AP; ThermoFisher, Waltham, MA, USA), betaine-homocysteine methyltransferase (BHMT) [43], phosphate cytidylyltransferase 1A, choline (PCYT1A) (ab109263, Abcam), choline kinase alpha (CHKA) (Invitrogen PA5-109529, ThermoFisher), phosphatidylethanolamine N-methyltransferase (PEMT) (Invitrogen PA5-42383; ThermoFisher), glyceraldehyde-3-phosphate dehydrogenase (GAPDH) (2118, Cell Signaling Technology, Danvers, MA, USA, VINCULIN (13901, Cell Signaling Technology), and ACTIN (A2066; Sigma-Aldrich, St. Louis, MO, USA). The extraction buffer contained protease inhibitors (Pierce, ThermoFisher) as well as phosphatase inhibitors (ThermoFisher) to preserve protein phosphorylation. Imaging and quantification of the blots was done using the Amersham Imager 600 (GE Healthcare Life Sciences, Marlborough, MA, USA) analysis software v1.0.0.

### 2.7. Statistical Analysis

Data from pregnant females were analysed by 2-factor ANOVA followed by Tukey post hoc analysis using GraphPad Prism software (version 6.01; GraphPad Software). Continuous variables from the embryos (e.g., CR lengths) were analysed using linear mixed models (MIXED procedure, SPSS Statistics version 22; IBM) specifying diet, maternal genotype (MG), and embryonic genotype (EG), and selected diet-genotype interactions (as determined by lowest Akaike information criterion (AIC) for the model) as fixed effects and including the litter as a random effect, followed by post hoc analysis corrected for multiple testing by Sidak. Categorical data (e.g., defects) were analysed by binary logistic regression with general linear mixed models using R software (version 4.0.3) [44] in RStudio (version 1.3.1093) [45] with package lme4 [46] followed by post hoc tests adjusted for multiple testing using the multivariate t method (mvt) with package emmeans [47]. Diet, maternal and embryonic genotypes, and selected diet-genotype interactions (as determined by lowest AIC) were specified as fixed effects and litter was included as a random effect. Individual females and embryos were used as the unit of analysis for calculations. Null (*Mthfd1S*^−/−^), necrotic, and damaged embryos were excluded from analysis. GraphPad Prism 6.01 was used to perform χ^2^ and Fisher’s exact analysis to compare embryonic genotype and sex distributions and for correlation analysis (computation of Pearson *r*). Values are presented as mean ± SEM. For all analyses, *p* ≤ 0.05 was considered significant; *p* ≤ 0.08 was considered a trend.

## 3. Results

### 3.1. Maternal Food Consumption and Weight Gain

There was no significant difference between CD and ChDD consumption over weeks 0 to 4 on the diets (2.61 ± 0.04 g/mouse/d vs. 2.65 ± 0.03 g/mouse/d; *p* = 0.38, unpaired *t*-test). WT and HET mice were housed together, so consumption by genotype could not be analysed. Pregnant mice consumed 32.50 ± 0.29 g of food between discovery of the plug and sacrifice; food consumption did not vary significantly with diet or genotype (*p*_genotype_ = 0.37, *p*_diet_ = 0.24, *p*_interaction_ = 0.054, 2-way ANOVA). There were no significant differences between the weights of the mice in the various diet/genotype groups at 0 and 4 weeks, in % weight gain after 4 weeks on diet, or during pregnancy (Table S2).

### 3.2. Maternal Body and Organ Weights at E10.5

Total body weights of pregnant HET mice at sacrifice tended to be lower than WT (*p*_genotype_ = 0.0632, *p*_diet_ = 0.69, *p*_interaction_ = 0.26, 2-way ANOVA) (Table S3). This is likely caused by the increased resorption rate that is usually observed in HET mice due to the lethality of the *Mthfd1S*^−/−^ (null) genotype [28,29] (Table S4). When the weight of the uterus was subtracted from body weight, to eliminate differences due to litter size, there was no significant difference in weights (*p*_genotype_ = 0.10, *p*_diet_ = 0.63, *p*_interaction_ = 0.27, 2-way ANOVA). The livers of HET mice were significantly lighter than WT (*p*_genotype_ = 0.0175, *p*_diet_ = 0.81, *p*_interaction_ = 0.52, 2-way ANOVA), although the difference was small, and there were no differences between groups by Tukey post-hoc. There were no significant effects of diet or genotype on spleen weight.

### 3.3. Fertility

Diet and genotype did not affect total number of implantation sites in the uterus at E10.5 (Table S4). There was a significant interaction between diet and genotype affecting the number of eggs released (*p*_genotype_ = 0.29, *p*_diet_ = 0.58, *p*_interaction_ = 0.0317, 2-way ANOVA), but there were no significant differences in post hoc tests. The ratio of implants/eggs released was not affected by diet or genotype, indicating that these factors did not affect pre-implantation loss. As expected, there was a significant increase in resorptions in HET mothers. There was no difference in the frequency of early resorptions (necrotic implantation sites), whereas the frequency of late resorptions (resorbing embryos in an intact decidua) was significantly increased because the null embryos die near E10.5 [27]. Resorption rate was not affected by diet.

### 3.4. Embryonic Genotype and Sex

In total, 98.7% of embryos were genotyped (Table S5). There was no significant deviation from expected ratios in litters of WT mothers. Consistent with the lethality of the null genotype, null embryos were significantly underrepresented in litters of HET mothers (*p* = 0.0014, chi-square), and all recovered null embryos were resorbing. ChDD did not alter genotype ratios in litters of WT or HET mice as compared to CD. There were no significant effects of diet, maternal genotype, or embryonic genotype on sex ratios of the embryos.

### 3.5. Embryo Characteristics at E10.5

#### 3.5.1. Developmental Delay 

Embryos were considered to be delayed if they were ≥ 1 day behind the most advanced embryo in the litter (examples in Figure S2). There was no significant difference in the ratio of males to females in delayed versus normal embryos (*p* = 0.33, Fisher’s exact test, 91.5% of delayed embryos were sexed); therefore, sex was not included in this analysis.

**Figure 1:**
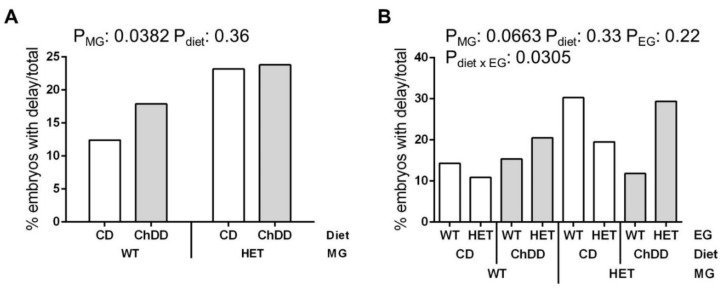
Effect of synthetase deficiency and low choline diet on incidence of developmental delay at E10.5. Values are % embryos with delay/total embryos evaluated. *n* = 33–78 embryos from 16–18 litters per group, analysed by binary logistic regression including litter as a random effect, mvt post-hoc. (**A**) Maternal effects, with embryo genotypes grouped. (**B**) Genotype and diet effects, including embryonic genotype. White bars: control diet (CD), grey bars: choline-deficient diet (ChDD). WT: wild type (*Mthfd1S*^+/+^); HET: heterozygous (*Mthfd1S*^+/−^); MG: maternal genotype; EG: embryonic genotype.

Since maternal genotype (MG) and diet are the only identifiable factors when planning a pregnancy, we examined these factors with and without including embryonic genotype in our analyses. When maternal factors were examined (genotype and diet), the incidence of delay was found to be significantly higher in embryos with HET mothers (Figure 1A). When both maternal and embryonic effects were examined, there was a trend towards an effect of maternal genotype on the incidence of delay, and there was a significant interaction between diet and embryonic genotype (EG) (Figure 1B, Table S6). ChDD appears to increase the incidence of delays among HET embryos, both when compared with CD HET embryos, and with ChDD WT embryos. However, there were no significant effects of these variables in post hoc comparisons. It is possible that the sample size is underpowered for this post hoc analysis, particularly for WT embryos from HET mothers (Table S6).

Embryonic crown-rump lengths (CR) mirrored the delay incidence in that embryos from HET mothers were significantly smaller than those from WT mothers (*p*_maternal genotype_ = 0.0149, *p*_diet_ = 0.94; Figure S3B). This maternal genotype effect was significant for both CD and ChDD diets (*p* = 0.0149 when compared to embryos with the same diet). Similarly, in the analysis including embryonic genotype, there were significant effects of maternal genotype, and the interaction of diet and embryonic genotype on crown-rump length (*p*_maternal genotype_ = 0.0151, *p*_diet_ = 0.86, *p*_embryonic genotype_ = 0.71, *p*_diet x embryonic genotype_ = 0.0104; Figure S3C). Embryos from HET mothers also had significantly fewer somites than those from WT mothers (*p*_maternal genotype_ = 0.0139, *p*_diet_ = 0.63, *p*_diet x maternal genotype_ = 0.34; Figure S4B). No other significant differences were observed for the somite numbers (Figure S4C).

#### 3.5.2. Defects

Defects observed in the embryos included open neural tubes, distortion of neural tube closure, reversed heart looping, and facial and forebrain malformations (examples in Figure S2). Defects were often accompanied by developmental delay (Table S6). The same types of defects were observed in all groups, only the incidence differed. There was no significant difference in the ratio of males to females in the defects versus normal embryos (*p* = 0.18 Fisher’s exact test, 93.0% of embryos with defects were sexed); therefore, sex was not included in the analysis.

**Figure 2:**
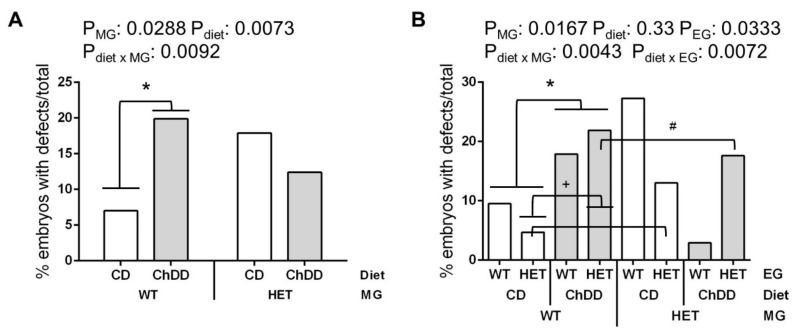
Effect of synthetase deficiency and low choline diet on incidence of defects at E10.5. Values are % embryos with defects/total embryos evaluated. *n* = 33–78 embryos from 16–18 litters per group, analysed by binary logistic regression including litter as a random effect, post hoc by mvt. (**A**) Maternal effects, with embryo genotypes grouped. * Significant increase in defects due to ChDD in the litters of WT mothers compared to CD WT mothers (*p* = 0.0259). (**B**) Genotype and diet effects, including embryonic genotype. * Significantly increased in ChDD WT mothers as compared to CD WT mothers (*p* = 0.0237). # Significantly increased in ChDD HET embryos as compared to CD HET (*p* = 0.0350). + Significantly increased in ChDD HET embryos with WT mothers as compared to CD HET embryos with WT mothers (*p* = 0.0030). White bars: CD, grey bars: ChDD.

Significant effects of both maternal genotype and diet were observed in the analysis of maternal factors (Figure 2A). In pairwise post hoc comparisons, there was a significant increase in defects due to ChDD in the litters of WT mothers compared to CD WT mothers.

There were significant effects of maternal genotype and embryonic genotype on the incidence of defects, as well as significant interactions between diet and the two genotypes (Figure 2B, Table S6). In pairwise post hoc comparisons of diet and maternal genotype, there was a significant increase in defects among embryos with ChDD WT mothers compared with CD WT mothers, and a trend towards increased defects among embryos with CD HET mothers as compared to CD WT mothers (*p* = 0.0571). In pairwise post hoc comparisons of diet and embryonic genotype, there was a significant increase in defects among ChDD HET embryos compared with CD HET embryos. In individual pairwise post hoc comparisons of all subgroups, HET embryos from WT mothers had an increase in defects in the ChDD group as compared to CD.

### 3.6. Maternal E10.5 Liver and Plasma Metabolites

#### 3.6.1. Choline and Methyl Metabolites in Maternal Liver

**Figure 3:**
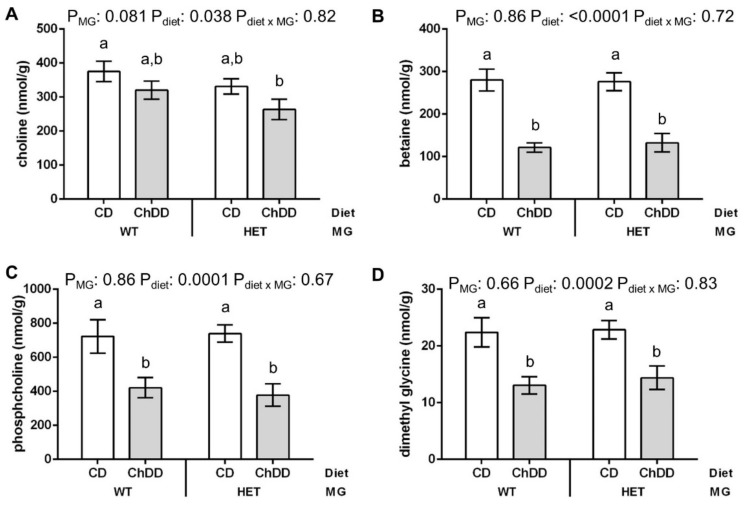
Effect of maternal synthetase activity and low choline diet on choline and methyl metabolites in maternal liver. Values are means ± SEM, *n* = 6 per group. Choline (**A**), betaine (**B**), phosphocholine (**C**), and dimethylglycine (**D**) were significantly lower in ChDD livers. Analysed by 2-way ANOVA; means without a common letter differ significantly by Tukey post hoc (*p* < 0.05). White bars: CD, grey bars: ChDD.

The concentrations of choline, betaine, phosphocholine (PCho, a choline storage form), and dimethylglycine were significantly lower in livers of mothers fed ChDD (Figure 3 and Table S7). There were no significant changes in the concentrations of methionine, GPC, PtdCho, sphingomyelin, lyso-PtdCho, SAM, SAH, or the SAM/SAH ratio (Table S7).

#### 3.6.2. Maternal Plasma and Liver Folate

There were no significant changes due to either maternal genotype or diet in total folate in maternal plasma or liver (Figure S5). The microbial folate assay does not evaluate specific folate forms, so we cannot rule out reduced amounts of specific folate derivatives.

#### 3.6.3. Phosphatidylcholine:Phosphatidylethanolamine in Maternal Liver 

**Figure 4:**
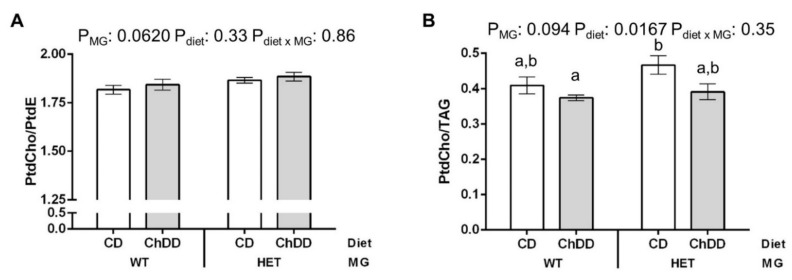
Effect of synthetase deficiency and low choline diet on PtdCho ratios in maternal liver. (**A**) Elevations in the PtdCho:PtdE ratio may indicate increased PtdCho synthesis by PEMT. (**B**) Decreases in the PtdCho:TAG ratio have been linked to decreased PtdCho synthesis via the CDP-choline pathway. Values are means ± SEM, *n* = 6 per group. Analysed by 2-way ANOVA; means without a common letter differ significantly by Tukey post hoc (*p* < 0.05). White bars: CD, grey bars: ChDD.

Lipid metabolites connected to PtdCho metabolism were measured. However, there were no significant differences in the concentrations of these metabolites (Table S8). The reduced availability of choline and related metabolites in the livers of ChDD-fed mothers suggests that the low choline diet could increase reliance on PtdCho synthesis from phosphatidylethanolamine (PtdE) by PEMT, rather than the choline-dependent CDP-choline (Kennedy) pathway. This can be evaluated by examining the PtdCho:PtdE ratio [48]. There was a near-significant trend towards elevated PtdCho:PtdE in the livers of HET mice (Figure 4A), which could indicate increased consumption of PtdE for PtdCho synthesis. There was also a significant decrease in the PtdCho:TAG ratio in ChDD mice (Figure 4B), which has been associated with decreased PtdCho synthesis via the CDP-choline pathway [49].

### 3.7. Immunoblotting

**Figure 5:**
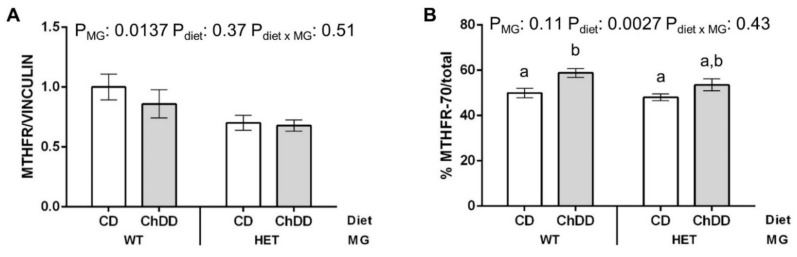
Effect of synthetase deficiency and low choline diet on maternal liver MTHFR expression. A representative blot is shown in Figure S6. (**A**) Total MTHFR expression, normalized to vinculin. (**B**) Expression of the more active 70 kDa isoform of MTHFR, as a percentage of total MTHFR. Values are means ± SEM, *n* = 6 per group. Analysed by 2-way ANOVA; means without a common letter differ significantly by Tukey post hoc (*p* < 0.05). White bars: CD, grey bars: ChDD.

MTHFD1, MTHFR, MTR, BHMT, PEMT, PCYT1A, and CHKA are important enzymes in this pathway in the maternal liver (Figure S1). To determine if the expression of these enzymes was affected by genotype or diet, protein levels were quantified by immunoblotting. There were no significant changes in expression, except for MTHFR (Figure 5 and Figure S6).

Three forms of MTHFR are detected by immunoblotting: the 70 kDa isoform, the less active phosphorylated 70 kDa isoform, and the 77 kDa isoform [50,51,52]. Total MTHFR expression was ~25% lower in livers of HET mothers, as compared to WT (Figure 5A), which may help to support nucleotide synthesis in these mice. In livers of ChDD mothers of both genotypes, the expression of the more active 70-kDa isoform of MTHFR, as % total MTHFR, increased ~15% (Figure 5B). A shift towards increased 70-kDa MTHFR may be a compensatory response to increased demand for methylTHF when the alternate methyl donor, betaine, is deficient.

## 4. Discussion

Choline is an under-recognized nutrient that is essential to support embryonic development [2,3,26]. Unfortunately, current choline intakes in most pregnant women and women of child-bearing age fail to meet the AI [22,23,24,25]. Furthermore, dietary requirements for choline may be increased by genetic variants, such as the common MTHFD1 R653Q polymorphism in the synthetase domain of this trifunctional enzyme [8,9,10,11,12,26]. In this study, we used the MTHFD1-synthetase-deficient mouse model to examine the hypothesis that pregnant 653QQ women may be more sensitive to choline deficiency with respect to reproductive outcomes. The low choline diet (ChDD) in this study contained ~1/3 of the choline in CD, a larger amount than the 1/8 diet we used in previous studies. In those studies, we demonstrated increases in heart defects and memory impairment due to choline deficiency during reproduction [21,53]. The choline content in this study was chosen to be proportional to the low end of reported choline intakes in North American women [24].

We observed that the incidence of developmental delays was significantly higher in the litters of HET mothers (23.5% versus 15.4%, Figure 1A). The incidence of defects increased in the litters of CD HET mothers as compared to CD WT (17.9% versus. 7.0%), and ChDD alone also significantly increased the incidence of defects in litters of WT mothers as compared to CD (19.9% versus 7.0%) (Figure 2A). Alterations in the synthesis of one-carbon folates and purines in HET mice [27] may affect the supply of key folate metabolites for the developing embryo and contribute to the increase in defects/delays in the litters of HET mothers. Diet-related alterations in maternal choline metabolites and PtdCho synthesis may also affect the supply of important metabolites for developing embryos, leading to increased defects in the litters of ChDD WT mothers. Although both ChDD and maternal HET genotype increased the risk of poor pregnancy outcomes, we did not see an additive effect of these factors.

There were significant interactions between diet and embryonic genotype that affected the incidence of both defects and delays. Embryos at this stage express MTHFD1 [54], MTHFR [55], PEMT, and the CDP-choline pathway [56,57]; disruptions of the embryos’ folate and PtdCho metabolism may contribute to defect risk. Embryos are dependent on their own MTHFD1-synthetase activity for proper growth and development, as shown by the lethality of the null genotype, and the impairment of de novo purine synthesis in HET embryonic fibroblasts [27]. Choline deficiency may lead to defects because of the altered synthesis of critical choline metabolites in maternal liver and/or embryonic tissues. The combination of reduced choline metabolism and decreased purine synthesis in rapidly proliferating embryo tissues may lead to the increased developmental delays and defects in ChDD HET embryos.

The combination of ChDD and embryonic HET genotype led to increased delay and defect susceptibility, whereas there was no additive effect of diet and maternal genotype. Ganz et al. [10] reported that the effects of MTHFD1 R653Q on choline metabolism were much more pronounced in lactating and non-pregnant women than in pregnant women. There may be changes in maternal choline metabolism during pregnancy that do not occur in embryonic choline metabolism. Their observations also suggest that the effects of choline deficiency on outcomes in HET mothers may be greater postnatally during lactation, which is known to be a choline-sensitive stage of brain development [17].

The liver is important for both folate and choline metabolism. The importance of hepatic choline metabolism is underscored by the report that liver imports choline from other tissues in response to choline deficiency [58]. This is consistent with our observation that ChdDD resulted in only a 17% decrease in maternal liver choline. The liver is the major site of expression of MTHFD1, and one of few organs where BHMT and PEMT are expressed. These enzymes interconnect folate and choline metabolism: BHMT and MTR both participate in the remethylation of homocysteine to methionine, using the choline metabolite betaine or methylTHF, respectively, as methyl donors, for generation of SAM. PEMT and the CDP-choline pathway both produce PtdCho, using SAM and choline respectively. PEMT is actually the primary consumer of SAM in this tissue [59]. Products of folate and choline metabolism in the maternal liver are transferred to embryos to support embryonic development [3]. PtdCho produced by PEMT in the maternal liver, rather than by the ubiquitous CDP-choline pathway, is preferentially sent to the developing foetus [60]. This partitioning may be important during development because the fatty acid chains in PtdCho from the two pathways differ. PEMT-produced PtdCho primarily contains longer chain polyunsaturated fatty acids, whereas the CDP-choline pathway mainly produces PtdCho containing saturated fatty acids [61]. In this study, maternal liver PtdCho concentrations were not affected by ChDD. PtdCho levels may be maintained by increasing synthesis, rather than by downregulating the breakdown of PtdCho, as GPC and lyso-PtdCho were also unchanged. In contrast, the concentration of phosphocholine, the primary choline storage form, decreased 45% in the liver of ChDD-fed mice. Decreased phosphocholine could indicate that the CDP-choline PtdCho synthesis pathway is disrupted, as it is formed in the first step of that pathway, or that phosphocholine stores are depleted to maintain PtdCho levels.

The maternal liver concentrations of betaine and dimethylglycine, which is produced when betaine is used as a methyl group donor, decreased 54% in the ChDD-fed mice. These findings suggest that either the production of betaine from choline is inhibited in favour of maintaining normal levels of PtdCho (by using choline in the CDP-choline pathway at the expense of other choline metabolites) or that betaine stores are depleted to support methylation reactions, such as PtdCho synthesis by PEMT. Betaine has other important roles in the cell, such as its role as an osmolyte. Therefore, reduced betaine concentrations may also impair embryonic development through mechanisms that are independent of homocysteine remethylation [62,63].

Total folate concentrations in maternal liver and plasma were measured to determine if the choline deficiency caused by ChDD was sufficient to cause folate deficiency, as has been reported in severely choline-deficient rats [15]. Neither plasma nor liver folates were affected by maternal diet or genotype (Figure S5); therefore the 3-fold decrease in choline was not sufficient to deplete total folate concentrations. Nonetheless, these results indicate that the diet effects in the embryos are not the result of total folate availability, although we cannot rule out reduced amounts of specific folate derivatives, e.g., changes in % methylTHF due to reduced MTHFR expression, or reduced % formylTHF in HET mice, as we previously reported [27].

The concentrations of methionine, SAM and SAH in the maternal ChDD liver did not differ from the CD liver, which suggests that on this folate-replete diet, there is sufficient folate-dependent (MTR) and betaine-dependent (BHMT) homocysteine remethylation to maintain methylation potential. The shift to a higher proportion of the more active 70 kDa isoform of MTHFR in these mice (Figure 5) may be a compensatory response to ChDD to produce methylTHF to maintain methionine and SAM, which would support PtdCho production by PEMT. The tendency of the PtdCho:PtdE ratio to rise in HET mice (Figure 4A), which suggests increased consumption of PtdE for PtdCho production, and the significant decrease in the PtdCho:TAG ratio in ChDD mice (Figure 4B), which has been associated with decreased PtdCho synthesis via the CDP-choline pathway [49], both suggest alterations in the balance of these PtdCho synthesis pathways. These shifts could affect embryonic development by altering the fatty acid composition of the PtdCho supplied to embryos. Large decreases in PtdCho:TAG have been linked to increased lipid droplet size [49], and both increases and decreases in PtdCho:PtdE have been associated with hepatic steatosis [48]. The PtdCho:PtdE ratio observed is within the 1.5–2 range for healthy murine liver [48], and the changes observed are quite small. However, these trends suggest that maternal liver health in the context of choline deficiency, particularly if folate intakes are also inadequate, should be considered in future studies.

## 5. Conclusions

Our findings suggest that choline deficiency may be of general concern during pregnancy due to the increase in defects that we observed in ChDD WT mothers. Additional risk may be conferred by the presence of the R653Q polymorphism. Further investigation is required to determine whether low choline intakes contribute to birth defects in humans, particularly in 653QQ women. An EAR for choline should be established to evaluate the prevalence of inadequate intakes.

## Data Availability

All relevant data are included in this article and Supplemental Materials.

## Supplementary Materials

The following are available online at https://www.mdpi.com/article/10.3390/nu14010127/s1: Figure S1: Choline and folate metabolism; Figure S2: Examples of E10.5 embryos with developmental delays and defects; Figure S3: Effect of maternal and embryonic synthetase activity and low choline diet on embryonic crown-rump length; Figure S4: Effect of maternal and embryonic synthetase activity and low choline diet on embryonic somite number; Figure S5: Effect of synthetase deficiency and low choline diet on total folate in plasma and liver; Figure S6: Representative immunoblot of maternal liver extracts; Table S1: Formulation of experimental diets; Table S2: Weights of WT and HET mice fed CD and ChDD; Table S3: Body and organ weights of WT and HET mice fed CD and ChDD; Table S4: Fertility of WT and HET mice fed CD and ChDD; Table S5: Embryonic genotype distributions; Table S6: Effect of maternal genotype, diet, and embryonic genotype on the incidence of delays and defects; Table S7: Hepatic choline and methylation metabolites in pregnant WT and HET mice fed CD and ChDD; Table S8: Lipid metabolites in pregnant WT and HET mice fed CD and ChDD.

## References

[1] Bailey L.B., Stover P.J., McNulty H., Fenech M.F., Gregory J.F., Mills J.L., Pfeiffer C.M., Fazili Z., Zhang M., Ueland P.M. (2015). Biomarkers of nutrition for development—Folate review. J. Nutr..

[2] Wallace T.C., Blusztajn J.K., Caudill M.A., Klatt K.C., Zeisel S.H. (2020). Choline: The neurocognitive essential nutrient of interest to obstetricians and gynecologists. J. Diet. Suppl..

[3] Korsmo H.W., Jiang X., Caudill M.A. (2019). Choline: Exploring the Growing Science on Its Benefits for Moms and Babies. Nutrients.

[4] Christensen K.E., Rohlicek C.V., Andelfinger G.U., Michaud J., Bigras J.L., Richter A., Mackenzie R.E., Rozen R. (2009). The MTHFD1 p.Arg653Gln variant alters enzyme function and increases risk for congenital heart defects. Hum. Mutat..

[5] Parle-McDermott A., Kirke P.N., Mills J.L., Molloy A.M., Cox C., O’Leary V.B., Pangilinan F., Conley M., Cleary L., Brody L.C. (2006). Confirmation of the R653Q polymorphism of the trifunctional C1-synthase enzyme as a maternal risk for neural tube defects in the Irish population. Eur. J. Hum. Genet..

[6] Parle-McDermott A., Pangilinan F., Mills J.L., Signore C.C., Molloy A.M., Cotter A., Conley M., Cox C., Kirke P.N., Scott J.M. (2005). A polymorphism in the MTHFD1 gene increases a mother’s risk of having an unexplained second trimester pregnancy loss. Mol. Hum. Reprod..

[7] Furness D.L., Fenech M.F., Khong Y.T., Romero R., Dekker G.A. (2008). One-carbon metabolism enzyme polymorphisms and uteroplacental insufficiency. Am. J. Obstet. Gynecol..

[8] Kohlmeier M., da Costa K.A., Fischer L.M., Zeisel S.H. (2005). Genetic variation of folate-mediated one-carbon transfer pathway predicts susceptibility to choline deficiency in humans. Proc. Natl. Acad. Sci. USA.

[9] Ivanov A., Nash-Barboza S., Hinkis S., Caudill M.A. (2009). Genetic variants in phosphatidylethanolamine N-methyltransferase and methylenetetrahydrofolate dehydrogenase influence biomarkers of choline metabolism when folate intake is restricted. J. Am. Diet. Assoc..

[10] Ganz A.B., Shields K., Fomin V.G., Lopez Y.S., Mohan S., Lovesky J., Chuang J.C., Ganti A., Carrier B., Yan J. (2016). Genetic impairments in folate enzymes increase dependence on dietary choline for phosphatidylcholine production at the expense of betaine synthesis. FASEB J..

[11] Ilozumba M.N., Cheng T.-Y.D., Neuhouser M.L., Miller J.W., Beresford S.A.A., Duggan D.J., Toriola A.T., Song X., Zheng Y., Bailey L.B. (2020). Associations between plasma choline metabolites and genetic polymorphisms in one-carbon metabolism in postmenopausal women: The women’s health initiative observational study. J. Nutr..

[12] Yan J., Winter L.B., Burns-Whitmore B., Vermeylen F., Caudill M.A. (2012). Plasma choline metabolites associate with metabolic stress among young overweight men in a genotype-specific manner. Nutr. Diabetes.

[13] Kim Y.I., Miller J.W., da Costa K.A., Nadeau M., Smith D., Selhub J., Zeisel S.H., Mason J.B. (1994). Severe folate deficiency causes secondary depletion of choline and phosphocholine in rat liver. J. Nutr..

[14] Jacob R.A., Jenden D.J., Allman-Farinelli M.A., Swendseid M.E. (1999). Folate nutriture alters choline status of women and men fed low choline diets. J. Nutr..

[15] Selhub J., Seyoum E., Pomfret E.A., Zeisel S.H. (1991). Effects of choline deficiency and methotrexate treatment upon liver folate content and distribution. Cancer Res..

[16] Christensen K.E., Wu Q., Wang X., Deng L., Caudill M.A., Rozen R. (2010). Steatosis in mice is associated with gender, folate intake, and expression of genes of one-carbon metabolism. J. Nutr..

[17] Derbyshire E., Obeid R. (2020). Choline, neurological development and brain function: A systematic review focusing on the first 1000 days. Nutrients.

[18] Shaw G.M., Carmichael S.L., Yang W., Selvin S., Schaffer D.M. (2004). Periconceptional dietary intake of choline and betaine and neural tube defects in offspring. Am. J. Epidemiol..

[19] Shaw G.M., Finnell R.H., Blom H.J., Carmichael S.L., Vollset S.E., Yang W., Ueland P.M. (2009). Choline and risk of neural tube defects in a folate-fortified population. Epidemiology.

[20] Fisher M.C., Zeisel S.H., Mar M.H., Sadler T.W. (2001). Inhibitors of choline uptake and metabolism cause developmental abnormalities in neurulating mouse embryos. Teratology.

[21] Chan J., Deng L., Mikael L.G., Yan J., Pickell L., Wu Q., Caudill M.A., Rozen R. (2010). Low dietary choline and low dietary riboflavin during pregnancy influence reproductive outcomes and heart development in mice. Am. J. Clin. Nutr..

[22] Lewis E.D., Subhan F.B., Bell R.C., McCargar L.J., Curtis J.M., Jacobs R.L., Field C.J. (2014). Estimation of choline intake from 24 h dietary intake recalls and contribution of egg and milk consumption to intake among pregnant and lactating women in Alberta. Br. J. Nutr..

[23] Masih S.P., Plumptre L., Ly A., Berger H., Lausman A.Y., Croxford R., Kim Y.-I., O’Connor D.L. (2015). Pregnant Canadian women achieve recommended intakes of one-carbon nutrients through prenatal supplementation but the supplement composition, including choline, requires reconsideration. J. Nutr..

[24] Wallace T.C., Fulgoni V.L. (2017). Usual choline intakes are associated with egg and protein food consumption in the United States. Nutrients.

[25] Bailey R.L., Pac S.G., Fulgoni V.L., Reidy K.C., Catalano P.M. (2019). Estimation of total usual dietary intakes of pregnant women in the United States. JAMA Netw. Open.

[26] Ganz A.B., Klatt K.C., Caudill M.A. (2017). Common genetic variants alter metabolism and influence dietary choline requirements. Nutrients.

[27] Christensen K.E., Deng L., Leung K.Y., Arning E., Bottiglieri T., Malysheva O.V., Caudill M.A., Krupenko N.I., Greene N.D., Jerome-Majewska L. (2013). A novel mouse model for genetic variation in 10-formyltetrahydrofolate synthetase exhibits disturbed purine synthesis with impacts on pregnancy and embryonic development. Hum. Mol. Genet..

[28] Christensen K.E., Hou W., Bahous R.H., Deng L., Malysheva O.V., Arning E., Bottiglieri T., Caudill M.A., Jerome-Majewska L.A., Rozen R. (2016). Moderate folic acid supplementation and MTHFD1-synthetase deficiency in mice, a model for the R653Q variant, result in embryonic defects and abnormal placental development. Am. J. Clin. Nutr..

[29] Christensen K.E., Bahous R.H., Hou W., Deng L., Malysheva O.V., Arning E., Bottiglieri T., Caudill M.A., Jerome-Majewska L.A., Rozen R. (2018). Low dietary folate interacts with MTHFD1 synthetase deficiency in mice, a model for the R653Q variant, to increase incidence of developmental delays and defects. J. Nutr..

[30] Li D., Pickell L., Liu Y., Wu Q., Cohn J.S., Rozen R. (2005). Maternal methylenetetrahydrofolate reductase deficiency and low dietary folate lead to adverse reproductive outcomes and congenital heart defects in mice. Am. J. Clin. Nutr..

[31] McFarlane L., Truong V., Palmer J.S., Wilhelm D. (2013). Novel PCR assay for determining the genetic sex of mice. Sex. Dev..

[32] Molloy A.M., Scott J.M. (1997). Microbiological assay for serum, plasma, and red cell folate using cryopreserved, microtiter plate method. Methods Enzymol..

[33] Bensadoun A., Weinstein D. (1976). Assay of proteins in the presence of interfering materials. Anal. Biochem..

[34] Koc H., Mar M.H., Ranasinghe A., Swenberg J.A., Zeisel S.H. (2002). Quantitation of choline and its metabolites in tissues and foods by liquid chromatography/electrospray ionization-isotope dilution mass spectrometry. Anal. Chem..

[35] Holm P.I., Ueland P.M., Kvalheim G., Lien E.A. (2003). Determination of choline, betaine, and dimethylglycine in plasma by a high-throughput method based on normal-phase chromatography-tandem mass spectrometry. Clin. Chem..

[36] Kim J.K., Harada K., Bamba T., Fukusaki E., Kobayashi A. (2005). Stable isotope dilution-based accurate comparative quantification of nitrogen-containing metabolites in *Arabidopsis thaliana* T87 cells using in vivo (15)N-isotope enrichment. Biosci. Biotechnol. Biochem..

[37] Jiang X., Yan J., West A.A., Perry C.A., Malysheva O.V., Devapatla S., Pressman E., Vermeylen F., Caudill M.A. (2012). Maternal choline intake alters the epigenetic state of fetal cortisol-regulating genes in humans. FASEB J..

[38] Folch J., Lees M., Sloane Stanley G.H. (1957). A simple method for the isolation and purification of total lipides from animal tissues. J. Biol. Chem..

[39] Abreu S., Solgadi A., Chaminade P. (2017). Optimization of normal phase chromatographic conditions for lipid analysis and comparison of associated detection techniques. J. Chromatogr. A.

[40] Lian J., van der Veen J.N., Watts R., Jacobs R.L., Lehner R. (2021). Carboxylesterase 1d (Ces1d) does not contribute to cholesteryl ester hydrolysis in the liver. J. Lipid Res..

[41] Gardam M.A., Mejia N.R., MacKenzie R.E. (1988). The NADP-dependent trifunctional methylenetetrahydrofolate dehydrogenase purified from mouse liver is immunologically distinct from the mouse NAD-dependent bifunctional enzyme. Biochem. Cell. Biol..

[42] Frosst P., Blom H.J., Milos R., Goyette P., Sheppard C.A., Matthews R.G., Boers G.J., den Heijer M., Kluijtmans L.A., van den Heuvel L.P. (1995). A candidate genetic risk factor for vascular disease: A common mutation in methylenetetrahydrofolate reductase. Nat. Genet..

[43] Rao P.V., Garrow T.A., John F., Garland D., Millian N.S., Zigler J.S. (1998). Betaine-homocysteine methyltransferase is a developmentally regulated enzyme crystallin in rhesus monkey lens. J. Biol. Chem..

[44] R Core Team R (2020). A Language and Environment for Statistical Computing.

[45] RStudio Team RStudio (2020). Integrated Development for R.

[46] Bates D., Mächler M., Bolker B., Walker S. (2015). Fitting linear mixed-effects models using lme4. J. Stat. Soft..

[47] Lenth R.V. (2020). Emmeans: Estimated Marginal Means, aka Least-Squares Means; R Package Version 1.5.3. https://CRAN.R-project.org/package=emmeans.

[48] Van der Veen J.N., Kennelly J.P., Wan S., Vance J.E., Vance D.E., Jacobs R.L. (2017). The critical role of phosphatidylcholine and phosphatidylethanolamine metabolism in health and disease. Biochim. Biophys. Acta Biomembranes.

[49] Moessinger C., Klizaite K., Steinhagen A., Philippou-Massier J., Shevchenko A., Hoch M., Ejsing C.S., Thiele C. (2014). Two different pathways of phosphatidylcholine synthesis, the Kennedy Pathway and the Lands Cycle, differentially regulate cellular triacylglycerol storage. BMC Cell Biol..

[50] Yamada K., Strahler J.R., Andrews P.C., Matthews R.G. (2005). Regulation of human methylenetetrahydrofolate reductase by phosphorylation. Proc. Natl. Acad. Sci. USA.

[51] Tran P., Leclerc D., Chan M., Pai A., Hiou-Tim F., Wu Q., Goyette P., Artigas C., Milos R., Rozen R. (2002). Multiple transcription start sites and alternative splicing in the methylenetetrahydrofolate reductase gene result in two enzyme isoforms. Mamm. Genome.

[52] Zheng Y., Ramsamooj S., Li Q., Johnson J.L., Yaron T.M., Sharra K., Cantley L.C. (2019). Regulation of folate and methionine metabolism by multisite phosphorylation of human methylenetetrahydrofolate reductase. Sci. Rep..

[53] Jadavji N.M., Deng L., Malysheva O., Caudill M.A., Rozen R. (2015). MTHFR deficiency or reduced intake of folate or choline in pregnant mice results in impaired short-term memory and increased apoptosis in the hippocampus of wild-type offspring. Neuroscience.

[54] Di Pietro E., Wang X.L., MacKenzie R.E. (2004). The expression of mitochondrial methylenetetrahydrofolate dehydrogenase-cyclohydrolase supports a role in rapid cell growth. Biochim. Biophys. Acta.

[55] Pickell L., Wu Q., Wang X.L., Leclerc D., Friedman H., Peterson A.C., Rozen R. (2011). Targeted insertion of two Mthfr promoters in mice reveals temporal- and tissue-specific regulation. Mamm. Genome.

[56] Wang L., Magdaleno S., Tabas I., Jackowski S. (2005). Early embryonic lethality in mice with targeted deletion of the CTP: Phosphocholine cytidylyltransferase alpha gene (Pcyt1a). Mol. Cell Biol..

[57] Wu G., Aoyama C., Young S.G., Vance D.E. (2008). Early embryonic lethality caused by disruption of the gene for choline kinase alpha, the first enzyme in phosphatidylcholine biosynthesis. J. Biol. Chem..

[58] Li Z., Agellon L.B., Vance D.E. (2007). Choline redistribution during adaptation to choline deprivation. J. Biol. Chem..

[59] Da Silva R.P., Kelly K.B., Al Rajabi A., Jacobs R.L. (2014). Novel insights on interactions between folate and lipid metabolism. Biofactors.

[60] Yan J., Jiang X., West A.A., Perry C.A., Malysheva O.V., Brenna J.T., Stabler S.P., Allen R.H., Gregory J.F., Caudill M.A. (2013). Pregnancy alters choline dynamics: Results of a randomized trial using stable isotope methodology in pregnant and nonpregnant women. Am. J. Clin. Nutr..

[61] DeLong C.J., Shen Y.J., Thomas M.J., Cui Z. (1999). Molecular distinction of phosphatidylcholine synthesis between the CDP-choline pathway and phosphatidylethanolamine methylation pathway. J. Biol. Chem..

[62] Zhao G., He F., Wu C., Li P., Li N., Deng J., Zhu G., Ren W., Peng Y. (2018). Betaine in inflammation: Mechanistic aspects and applications. Front. Immunol..

[63] Tscherner A.K., Macaulay A.D., Ortman C.S., Baltz J.M. (2021). Initiation of cell volume regulation and unique cell volume regulatory mechanisms in mammalian oocytes and embryos. J. Cell Physiol..

