# Supplementary material for: Mild Choline Deficiency and MTHFD1 Synthetase Deficiency Interact to Increase Incidence of Developmental Delays and Defects in Mice
Source: Nutrients. 2021 Dec 28;14(1):127. doi: 10.3390/nu14010127 (PMC8747146; doi:10.3390/nu14010127)
Supplement: Supplementary file 1 [file nutrients-14-00127-s001.zip › Christensen et al 2021 Supplemental Figures.pdf]

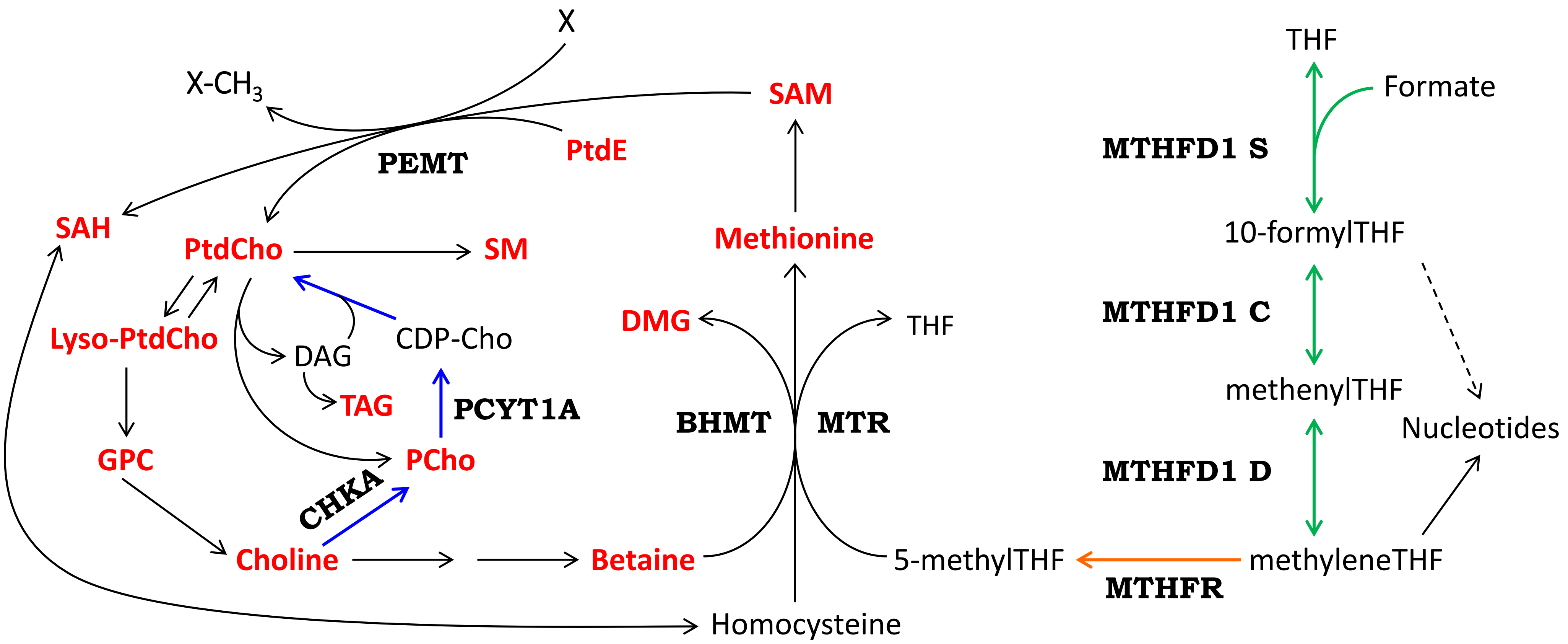

Figure S1. Choline and folate metabolism. Choline (left) and folate (right) metabolism intersect through the detoxification of homocysteine, which produces methionine via BHMT or MTR, respectively. Methionine is used to make SAM, a major methyl donor for numerous reactions including DNA methylation, and PtdCho synthesis from PtdE by PEMT in liver, which requires 3 molecules of SAM for each molecule of PtdCho produced. PtdCho is also synthesized by the CDP-choline pathway (blue arrows), and can in turn be broken down to supply choline. MTHFD1 (green arrows) supplies the methyleneTHF substrate of MTHFR (orange arrow) to produce 5-methylTHF for homocysteine detoxification. The R653Q variant is located in the synthetase domain of MTHFD1 (MTHFD1 S). Key enzymes assessed by immunoblotting are labeled. Measured metabolites are in red.

Abbreviations: BHMT, betaine-homocysteine methyltransferase; CDP-Cho, cytidine 5'-diphosphocholine; CHKA, choline kinase alpha; DAG, diacylglycerol; DMG, dimethylglycine; GPC, glycerophosphocholine; MTHFD1, methyleneTHF dehydrogenase (D)-methenylTHF cyclohydrolase (C)-10-formylTHF synthetase (S); MTHFR, methyleneTHF reductase; MTR, methionine synthase; PCho, phosphocholine; PCYT1A, phosphate cytidylyltransferase 1A, choline; PEMT, phosphatidylethanolamine N-methyltransferase; PtdCho, phosphatidylcholine; PtdE, phosphatidylethanolamine; SAH, S-adenosylhomocysteine; SAM, S-adenosylmethionine; SM, sphingomyelin; TAG, triacylglycerol; THF, tetrahydrofolate.

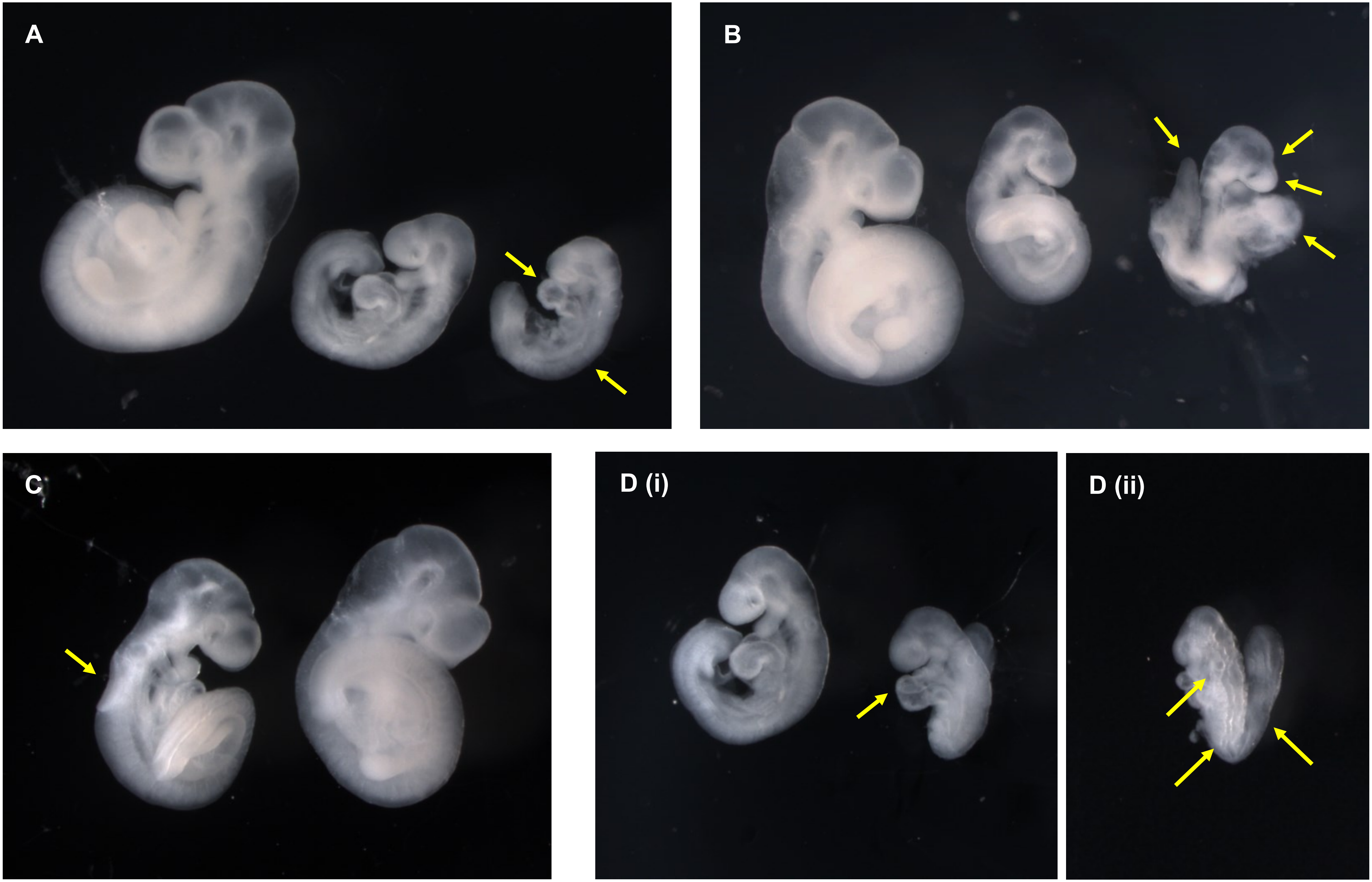

Figure S2: Examples of E10.5 embryos with developmental delays and defects. All images are at the same magnification. Location of defects is indicated by arrows. Open neural tubes at the anterior and posterior neuropore could be a delay effect and these were not counted as defects when they were consistent with the developmental markers evaluated. **A)** Littermates, from left to right: normal, delayed, and delayed with defects (abnormal heart, distorted neural tube closure). **B)** Littermates, from left to right: normal, delayed, and delayed with defects (abnormal face, forebrain, heart, and tail development, and failure to turn). **C)** On left, embryo with open neural tube below the otic pit, next to normal littermate on right. **Di)** On right, delayed embryo with reversed heart looping and distorted neural tube closure, shown with an embryo at the same developmental stage. A view of the distortion of the neural tube is shown in panel Dii.

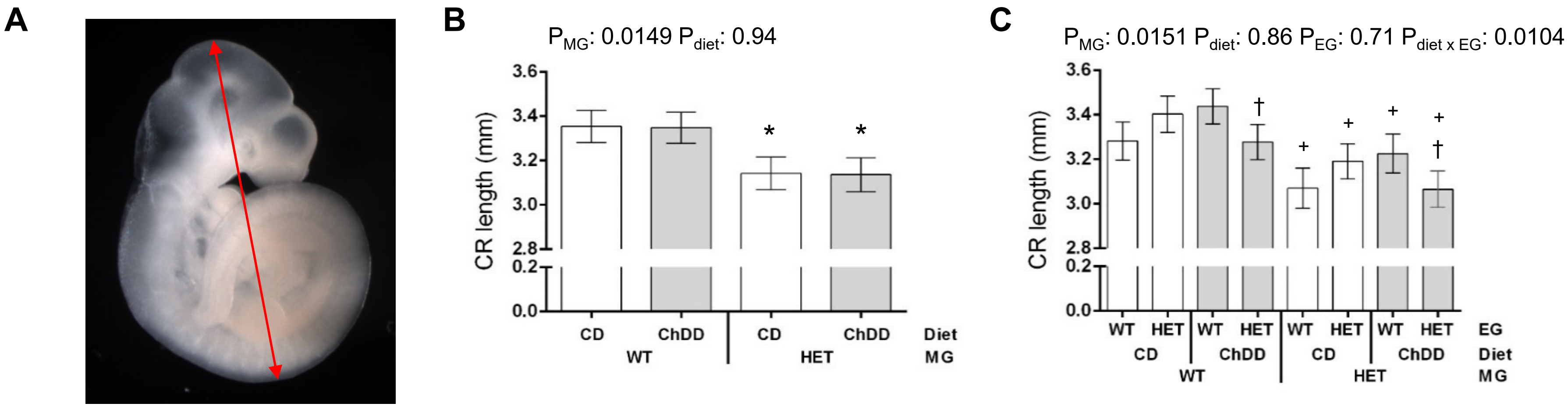

Figure S3: Effect of maternal and embryonic synthetase activity and low choline diet on embryonic crown-rump (CR) length. 16-18 litters per group. Values are estimated marginal means ± SEM. Analysed by linear mixed models including litter as a random effect, Sidak post-hoc. **A)** Crown-rump length is the distance from the top of the head to the rump, as shown by the red arrow. **B)** Maternal effects, embryo genotypes grouped. n = 92-142 embryos per group. * Embryos from HET mothers were significantly smaller than from WT mothers with the same diet, P=0.0149. **C)** Genotype and diet effects, including embryonic genotype. n = 32-72 embryos per group. + Embryos from HET mothers were significantly smaller than embryos from WT mothers with the same diet and embryonic genotype, P=0.0151; † ChDD HET embryos were significantly different from ChDD WT embryos with same maternal genotype. White bars: CD, grey bars: ChDD.

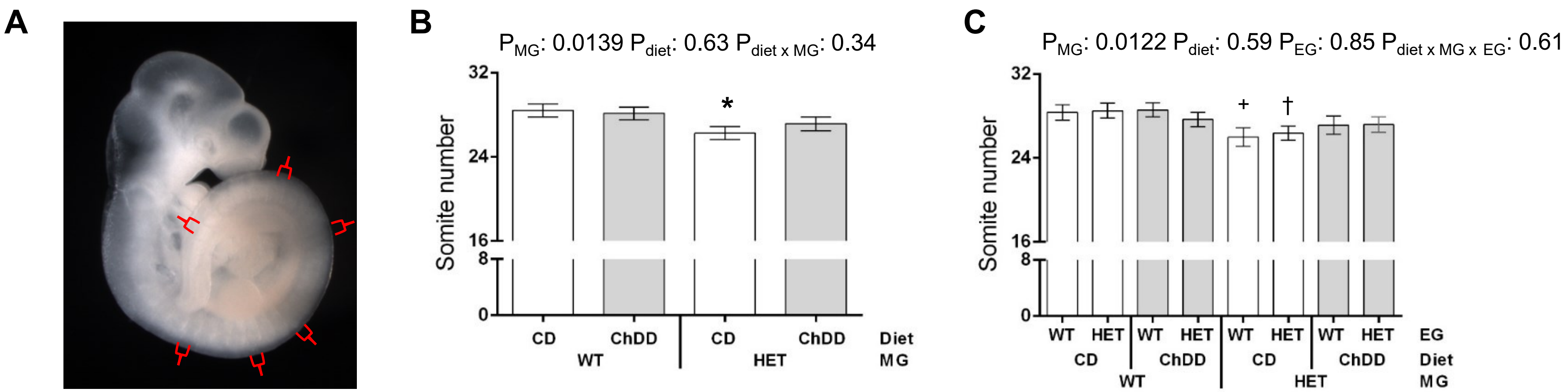

Figure S4: Effect of maternal and embryonic synthetase activity and low choline diet on embryonic somite number. 16-18 litters per group. Values are estimated marginal means ± SEM. Analysed by linear mixed models including litter as a random effect, Sidak post-hoc. **A)** Somites were counted as an indicator of developmental stage. The red brackets show examples of where the somites are found. **B)** Maternal effects, embryos grouped. n = 93-140 embryos per group. * Embryos from CD HET mothers were significantly different from embryos with CD WT mothers (P=0.0159). **C)** Genotype and diet effects, including embryonic genotype. n = 31-73 embryos per group. + CD WT embryos from HET mothers were significantly different from CD WT embryos with WT mothers (P=0.0413); † CD HET embryos from HET mothers were significantly different from CD HET embryos with WT mothers (P=0.0306). White bars: CD, grey bars: ChDD.

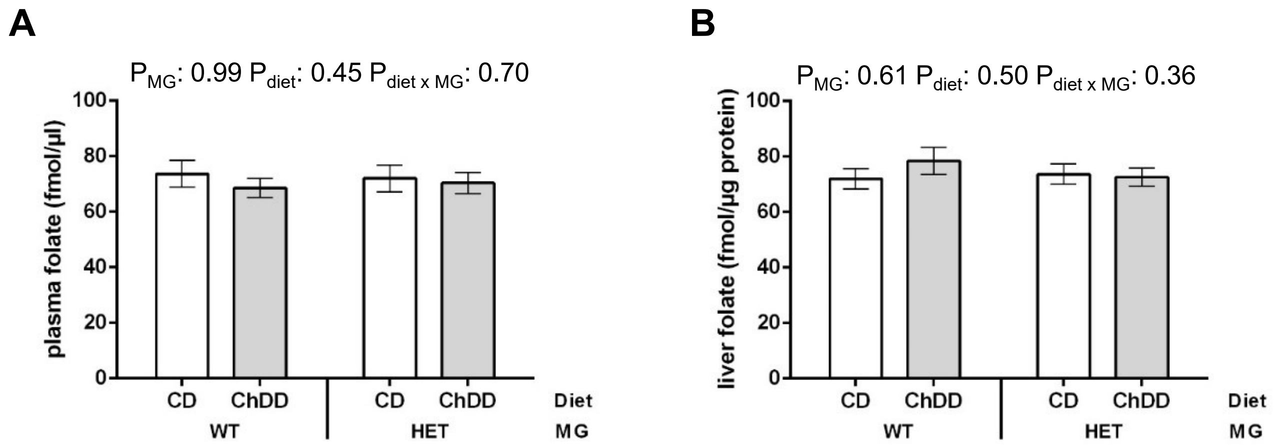

Figure S5: Effect of synthetase deficiency and low choline diet on total folate in **(A)** plasma and **(B)** liver. Values are means ± SEM, n=5-6 per group, 5 replicates per sample. Analysed by 2-way ANOVA. White bars: CD, grey bars: ChDD.

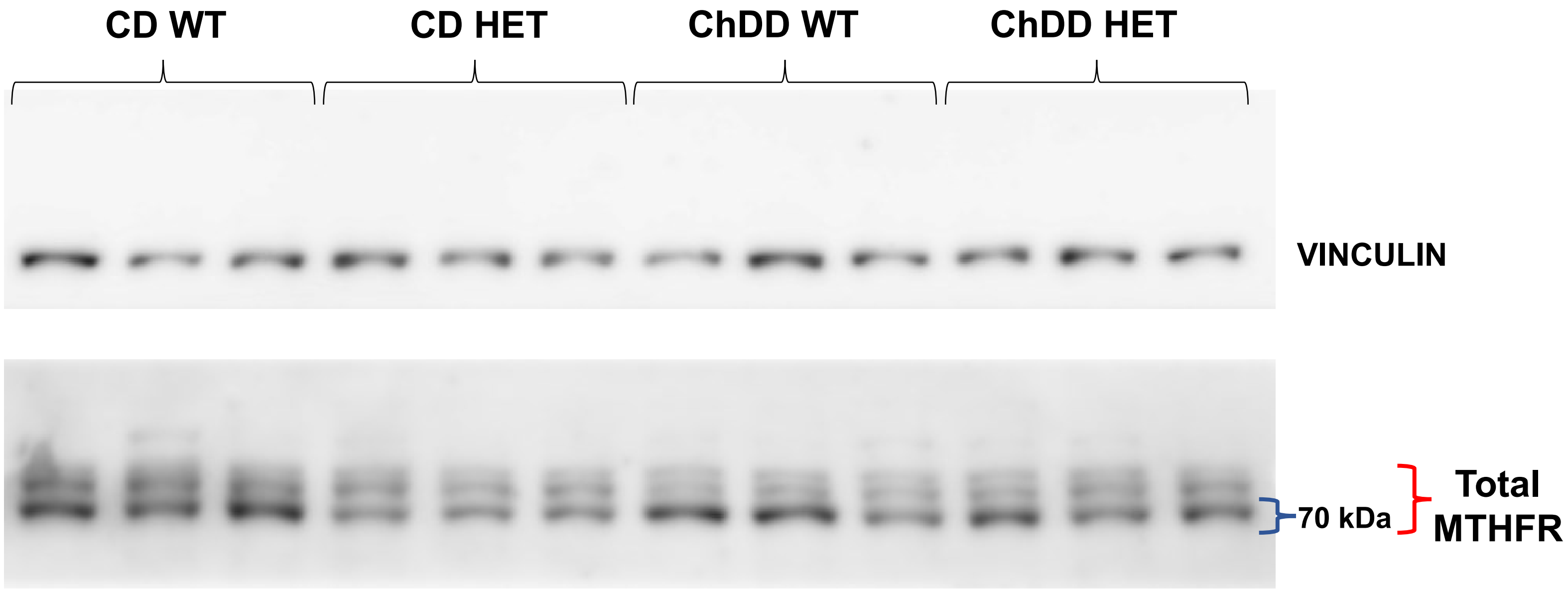

Figure S6: Representative immunoblot of maternal liver extracts. Immunoreactive MTHFR was normalized to the VINCULIN loading control. Shifts in the relative proportions of the MTHFR isoforms affect MTHFR activity because the non-phosphorylated 70 kDa MTHFR isoform has greater MTHFR activity [50-52].
