# Supplementary material for: Mild Choline Deficiency and MTHFD1 Synthetase Deficiency Interact to Increase Incidence of Developmental Delays and Defects in Mice
Source: Nutrients. 2021 Dec 28;14(1):127. doi: 10.3390/nu14010127 (PMC8747146; doi:10.3390/nu14010127)
Supplement: Supplementary file 1 [file nutrients-14-00127-s001.zip › Christensen et al 2021 Supplemental Tables.pdf]

Online Supplemental Material

Table S1: Formulation of experimental diets[1]

Diet Composition (g/kg)	CD (TD.01369) (2.5 g/kg choline bitartrate)	ChDD (TD.180221) (0.8 mg/kg choline bitartrate)
L-alanine	3.5	3.5
L-arginine HCl	12.1	12.1
L-asparagine	6.0	6.0
L-aspartic acid	3.5	3.5
L-cystine	3.5	3.5
L-glutamic acid	40.0	40.0
Glycine	23.3	23.3
L-histidine HCl, monohydrate	4.5	4.5
L-isoleucine	8.2	8.2
L-leucine	11.1	11.1
L-lysine HCl	18.0	18.0
L-methionine	3.3	3.3
L-phenylalanine	7.5	7.5
L-proline	3.5	3.5
L-serine	3.5	3.5
L-threonine	8.2	8.2
L-tryptophan	1.8	1.8
L-tyrosine	5.0	5.0
L-valine	8.2	8.2
Sucrose	349.53	351.13
Corn starch	150.0	150.0
Maltodextrin	150.0	150.0
Soybean oil	80.0	80.0
Cellulose	30.0	30.0
Mineral Mix, AIN-93M-MX	35.0	35.0
Calcium phosphate, monobasic, monohydrate	8.2	8.2
Succinylsulfathiazole	10.0	10.0
Vitamin Mix, AIN-93-VX [2]	10.0	10.0
Choline bitartrate	2.5	0.8
Vitamin K, menadione sodium bisulfite	0.05	0.05
tert-Butylhydroquinone (TBHQ) antioxidant	0.02	0.02
Orange food color	-	0.1

[1] Diet formulation is based on TD.99366 (a standard amino acid defined diet; Envigo). Vitamin, mineral and choline content is based on recommendations for AIN-93G (Reeves PG. *J Nutr* 1997; 127:838S-41S); amino acid content is based on Rogers QR and Harper AE. *J Nutr* 1965; 87:267-73. Methionine content is lower than in Rogers and Harper; however, total methionine + cysteine content exceeds the minimum stated in the NRC guidelines (National Research Council. Nutrient Requirements of Laboratory Animals: 4th ed. Washington, DC: National Academies Press (US), 1995).

[2] This amount Vitamin Mix AIN-93-VX provides a final concentration of 2 mg/kg folic acid to the diet.

Table S2: Weights of WT and HET mice fed CD and ChDD (mean±SEM)

						2-way ANOVA		
**Timepoint**	**Genotype**	**Diet**	**n**	**Weight[1] (g)**	**% weight gain**	**P genotype**	**P diet**	**P interaction**
t=0	WT	CD	16	15.92±0.28		0.89	0.19	0.90
		ChDD	18	16.43±0.36				
	HET	CD	18	16.01±0.37				
		ChDD	16	16.43±0.40				
t=4 weeks	WT	CD	16	19.76±0.29		0.22	0.91	0.55
		ChDD	18	19.96±0.30				
	HET	CD	18	19.59±0.25				
		ChDD	16	19.46±0.25				
0-4 weeks	WT	CD	16		24.52±2.11	0.30	0.14	0.76
		ChDD	18		22.02±1.92			
	HET	CD	18		22.97±1.82			
		ChDD	16		19.16±2.53			
Mating-E10.5[1]	WT	CD	16		9.90±0.99	0.50	0.08	0.17
		ChDD	18		9.53±0.73			
	HET	CD	18		10.59±0.82			
		ChDD	16		7.57±1.26			

[1] Weight gain during pregnancy was determined using (total body weight at E10.5 – uterus weight) to account for differences in litter sizes

Table S3: Body and organ weights of WT and HET mice fed CD and ChDD (mean±SEM)

	Genotype	Diet	n	mean ± SEM	2-way ANOVA P genotype	P diet	P interaction
Total body weight (g)	WT	CD	16	23.52±0.31	0.0632	0.69	0.26
		ChDD	18	23.78±0.35			
	HET	CD	18	23.25±0.40			
		ChDD	16	22.72±0.32			
Body weight - uterus (g)	WT	CD	16	22.68±0.29	0.10	0.63	0.27
		ChDD	18	22.89±0.34			
	HET	CD	18	22.49±0.38			
		ChDD	16	21.95±0.31			
Liver (% body weight-uterus)	WT	CD	16	6.45±0.06	**0.0175**	0.81	0.52
		ChDD	18	6.42±0.08			
	HET	CD	18	6.17±0.14			
		ChDD	16	6.25±0.04			
Spleen (% body weight-uterus)	WT	CD	16	0.655±0.019	0.22	0.14	0.44
		ChDD	18	0.691±0.013			
	HET	CD	18	0.647±0.017			
		ChDD	16	0.659±0.015			

Table S4: Fertility of WT and HET mice fed CD and ChDD (mean±SEM)

					2-way ANOVA		
	**Genotype**	**Diet**	**n**		**P Genotype**	**P Diet**	**P interaction**
N implants	WT	CD	16	10.2±0.5	0.81	0.81	0.43
		ChDD	18	10.4±0.6			
	HET	CD	18	10.7±0.4			
		ChDD	16	10.2±0.4			
N eggs released	WT	CD	16	11.9±0.6	0.29	0.58	0.0317
		ChDD	18	12.8±0.5			
	HET	CD	18	12.6±0.6			
		ChDD	16	11.1±0.3			
implants/eggs	WT	CD	16	0.87±0.05	0.29	0.86	0.28
		ChDD	18	0.83±0.05			
	HET	CD	18	0.87±0.04			
		ChDD	16	0.92±0.02			
N resorptions	WT	CD	16	2.2±0.5	<0.0001	0.45	0.40
		ChDD	18	2.2±0.3			
	HET	CD	18	4.7±0.4			
		ChDD	16	4.0±0.5			
% resorptions (% of total implants)	WT	CD	16	20.4±4.0	<0.0001	0.57	0.42
		ChDD	18	21.3±2.9			
	HET	CD	18	44.0±3.3			
		ChDD	16	39.0±4.3			
% early resorb (% of total implants)	WT	CD	16	10.2±2.2	0.50	0.59	0.53
		ChDD	18	7.7±1.9			
	HET	CD	18	10.3±2.1			
		ChDD	16	10.5±2.2			
% late resorb (% of total implants)	WT	CD	16	10.2±3.1	<0.0001	0.79	0.23
		ChDD	18	13.6±3.4			
	HET	CD	18	33.7±3.5			
		ChDD	16	28.5±4.3			

Table S5: Embryonic genotype distributions

Maternal Genotype	Diet	Embryonic Genotype WT	HET	NULL	Total [a]	χ^2 vs expected ***P***
WT	CD	63	64	-	127	0.93
	ChDD	78	73	-	151	0.68
HET	CD	33	77	21	131	0.0442
	ChDD	34	68	17	119	0.0262

[a] excludes embryos that could not be genotyped: (2 CD WT, 2 CD HET, 3 ChDD HET).

Table S6: Effect of maternal genotype, diet, and embryonic genotype on the incidence of delays and defects

	Maternal Genotype	Diet	n litters	Embryo Genotype	n embryos evaluated	n affected embryos	n affected litters
Delay[1]	WT	CD	16	WT	63	9	10
				HET	64	7	
		ChDD	18	WT	78	12	13
				HET	73	15	
	HET	CD	18	WT	33	10	12
				HET	77	15	
		ChDD	16	WT	34	4	14
				HET	68	20	
Defects[2]	WT	CD	16	WT	63	6	7
				HET	64	3	
		ChDD	18	WT	78	14	14
				HET	73	16[a]	
	HET	CD	18	WT	33	9	11
				HET	77	10	
		ChDD	16	WT	34	1	10
				HET	68	12	

[1] Delay: $P_{maternal\ genotype} = 0.0663$, $P_{diet} = 0.33$, $P_{embryonic\ genotype} = 0.22$, $P_{diet\ x\ embryonic\ genotype} = 0.0305$; binary logistic regression including litter as a random effect.

41 delayed embryos also had defects (out of 92)

[2] Defects: $P_{maternal\ genotype} = 0.0167$, $P_{diet} = 0.33$, $P_{embryonic\ genotype} = 0.0333$, $P_{diet\ x\ maternal\ genotype} = 0.0043$, $P_{diet\ x\ embryonic\ genotype} = 0.0072$; binary logistic regression including litter as a random effect.

41 embryos with defects also were delayed (out of 71)

[a] significantly different from CD with same maternal/embryonic genotypes (P=0.0030, post hoc by mvt)

Table S7: Hepatic choline and methylation metabolites in pregnant *WT* and *HET* mice fed CD and ChDD (n = 6/group, mean±SEM)

Metabolite	Genotype	Diet	Concentration (nmol/g)	2-way ANOVA P Genotype	P Diet	P interaction
Choline	WT	CD	375.31±30.08	0.08	**0.0380**	0.82
		ChDD	320.43±26.31			
	HET	CD	331.06±22.63			
		ChDD	263.78±30.23			
Betaine	WT	CD	280.01±25.94	0.86	**< 0.0001**	0.72
		ChDD	121.50±11.15			
	HET	CD	276.10±20.96			
		ChDD	132.56±21.50			
Phosphocholine	WT	CD	722.70±98.27	0.86	**0.0001**	0.67
		ChDD	421.42±59.43			
	HET	CD	739.88±51.48			
		ChDD	377.99±65.82			
Dimethylglycine	WT	CD	22.39±2.57	0.65	**0.0002**	0.83
		ChDD	13.06±1.51			
	HET	CD	22.87±1.63			
		ChDD	14.39±2.07			
Methionine	WT	CD	78.65±5.64	0.88	0.88	0.77
		ChDD	80.94±6.60			
	HET	CD	80.93±2.84			
		ChDD	80.22±4.81			
Glycerophospho-choline	WT	CD	439.77±65.05	0.87	0.50	0.33
		ChDD	369.58±35.90			
	HET	CD	405.58±24.81			
		ChDD	417.99±28.41			
Phosphatidyl-choline	WT	CD	24617±1876	0.93	0.73	0.99
		ChDD	24211±788			
	HET	CD	24734±900			
		ChDD	24301±773			
Sphingomyelin	WT	CD	2148±117	0.20	0.73	*0.0612*
		ChDD	2023±48			
	HET	CD	1895±59			
		ChDD	2073±62			
Lysophosphatidyl-choline	WT	CD	491.28±36.19	0.39	0.93	0.75
		ChDD	480.06±18.54			
	HET	CD	506.11±25.85			
		ChDD	512.39±22.73			
S-adenosyl-methionine	WT	CD	53.62±4.32	0.29	0.59	0.13
		ChDD	48.55±3.31			
	HET	CD	40.30±5.89			
		ChDD	50.89±6.06			
S-adenosyl-homocysteine	WT	CD	37.65±2.05	*0.0574*	0.11	0.23
		ChDD	36.20±1.41			
	HET	CD	48.58±5.94			
		ChDD	38.82±1.91			
SAM/SAH	WT	CD	1.455±0.162	0.15	0.37	0.14
		ChDD	1.347±0.089			
	HET	CD	0.937±0.199			
		ChDD	1.355±0.207			

Table S8: Lipid metabolites in pregnant *WT* and *HET* mice fed CD and ChDD (n = 6/group, mean±SEM)

				2-way ANOVA		
**Metabolite**	**Genotype**	**Diet**	**Concentration (µg/mg ptn)**	**P Genotype**	**P Diet**	**P interaction**
Triacylglycerol (TAG)	WT	CD	180.2±8.3	0.23	0.15	0.64
		ChDD	191.7±6.2			
	HET	CD	160.3±12.1			
		ChDD	182.8±16.6			
Free cholesterol	WT	CD	27.10±0.46	0.38	0.51	0.85
		ChDD	26.52±0.60			
	HET	CD	27.57±0.29			
		ChDD	27.25±1.05			
Phosphatidyl-ethanolamine (PtdE)	WT	CD	40.10±1.03	0.38	0.23	0.75
		ChDD	38.83±0.99			
	HET	CD	39.31±1.20			
		ChDD	37.14±2.05			
Phosphatidyl-choline (PtdCho)	WT	CD	72.76±1.23	0.79	0.35	0.63
		ChDD	71.64±2.50			
	HET	CD	73.29±1.98			
		ChDD	69.82±3.35			
PtdCho:PtdE	WT	CD	1.817±0.023	*0.0620*	0.33	0.86
		ChDD	1.844±0.028			
	HET	CD	1.866±0.015			
		ChDD	1.885±0.023			
PtdCho:TAG	WT	CD	0.409±0.024	0.094	**0.0167**	0.35
		ChDD	0.374±0.008			
	HET	CD	0.467±0.026 [a]			
		ChDD	0.391±0.022			

[a] significantly different from WT ChDD (P=0.0272, Tukey post hoc), P=0.0862 in comparison with HET ChDD.
